# Detection of *Legionella* spp. from Domestic Water in the Prefecture of Arta, Greece

**DOI:** 10.1155/2014/407385

**Published:** 2014-03-12

**Authors:** Dimitra Dimitriadi, Emmanuel Velonakis

**Affiliations:** ^1^Department of Microbiology, National School of Public Health, 196 Alexandras Avenue, 115 21 Athens, Greece; ^2^Central Public Health Laboratory, 34 Alexandrou Fleming, Vari, 16672 Attika, Greece

## Abstract

The aim of this research was the isolation of *Legionella* spp. from domestic water supply networks in the Prefecture of Arta. A total of 100 water samples, from 25 houses, were collected. Half of the samples concerned the cold water and half the hot water supply. Purpose was to detect colonization of the water networks with *Legionella* spp. >500 cfu/L by using the method of filtration (ISO 11731). Out of 100 samples, 6 samples from 3 houses were positive for *Legionella* spp. *Legionella pneumophila* serogroup 2–14 was isolated in 5 of 6 samples, whereas in the sixth sample *Legionella anisa* was identified. Only three of the samples had residual chloride over 0.2 mg/L, rate which is necessary for potable water, according to the Greek hygienic practice. Concerning the temperature of hot water, the mean temperature of the negative for *Legionella* samples was higher compared to the mean temperature of the positive for *Legionella* samples (49.9°C versus 45.5°C). It is estimated that there is risk of infection through the use of showers. The low concentration of chloride and the temperature, which was found within the limits favorable to developing *Legionella* spp. (20–45°C), provide fertile ground for proliferation of the bacteria.

## 1. Introduction

In 1976, an outbreak of severe pneumonia among the participants of the American Legion Convention in Philadelphia led to the description of Legionnaires' disease. The disease was found to be caused by the bacterium *Legionella pneumophila* (*Legionella* after the legionnaires' who were infected at the convention, *pneumophila* meaning “lung-loving”), belonging to the family Legionellaceae. *Legionella* has been retrospectively identified as the cause of outbreaks of Legionnaires' disease since 1947 [1].

*Legionellae* are ubiquitous in natural water environments worldwide. They are transmitted through the water supply networks that serve both public and private properties.

*Legionella* is a serious pathogen in health-care facilities affecting mainly immunocompromised patients. The bacterium can also cause community-acquired pneumonia, which involves a high rate of hospital admission. Legionnaires' disease is also recognized as a major form of travel-associated pneumonia, and about 20% of the cases of legionellosis detected in Europe are considered to be related to travel; these cases present a particular problem because of difficulties in identifying the source of infection. Although *Legionella* is a well-recognized problem in developed countries, data are scarce from developing countries, and the problem of *Legionella* is probably underestimated [2].

The bacterium is transmitted through inhalation of droplets containing *Legionellae, *commonly referred to as aerosols. Aerosols with diameter less than 5 *μ*m are more likely to cause severe illness, because they can enter deeply the respiratory system [3]. *Legionella* infections have been associated with sources at distances up to 3.2 Km. Recent evidence suggests that infection may be possible at even longer distances. Legionnaires' disease, however, cannot be transmitted from person to person [4].

*Legionella* causes a collection of infections, which can range in severity from a mild febrile illness (Pontiac fever) to a potentially fatal form of pneumonia (Legionnaires' disease).

The bacterium can survive in a range of environmental conditions. It has been isolated from environmental sources with pH ranging from 2.7 to 8.3. Temperatures between 20 and 45°C are ideal for the proliferation of the organism, with an optimal temperature range of 35–45°C. As the temperature falls, reproductive rates decrease and there is little or no increase in bacteria number when the temperature is below 20°C. *Legionella *can survive for long periods at a low temperature and then proliferate when the temperature increases. At the temperature of 66°C *Legionella* dies within two minutes and at temperature above 70°C they are destroyed instantly [2].

The presence of other microorganisms such as amoeba and algae supports the proliferation of the bacteria. Other favorable conditions are considered to be the presence of saline, rust, and sludge as well as the formation of biofilms [2].

Positive correlations regarding contamination with *Legionella* spp. have been observed in association with the type of heater, whether hot water is provided by either oil or gas heater or an electric heater, as well as with the age of the district and the water heating system [5].

The first evidence that potable water might be associated with legionellosis was reported by Tobin et al. who obtained two *Legionella pneumophila* serogroup 6 isolates from patients in a renal graft unit and isolated similar strains from shower-bath mixers in the same unit [6].

There are numerous reports of colonization of water system in large buildings such as hospitals, nursing homes, and hotels and the current knowledge about the epidemiology of legionellosis is based mainly on data gathered from studies of outbreaks. On the other hand little research has been done concerning the growth of *Legionella *in small residents, the factors that are associated with the contamination, and the epidemiology of the sporadically occurring cases of community-acquired legionellosis [7].

In an investigation in Catalonia *Legionella* spp. was isolated in 8% of the water samples from the houses of 124 patients with Legionnaires' disease, with predomination of serogroup 1 [8]. In another study in Italy, concerning 6 towns, *Legionella* spp. was isolated in 22.6% of domestic water samples, with predomination of serogroup 2–14 [7]. In previous studies in Finland and Germany, the occurrence of *Legionellae* was similar (30% and 26%, resp.,), with predomination of serogroup 2–14 [9, 10]. According to research conducted by Velonakis et al. in Greece, *Legionella* spp. was isolated from other Prefectures as well (unpublished data).

Taking into consideration the lack of data concerning the colonization of *Legionella *spp. in domestic level, we conducted a research in order to estimate the frequency of *Legionella* isolation from small residents and to identify the factors promoting the growth of the bacterium.

The aim of the present research was “the detection of *Legionella* spp. from domestic water supply networks in the Prefecture of Arta.” Special aims of the research were the estimation of sanitary condition of the water supply network of domestic water in the Prefecture of Arta, concerning the detection of *Legionella* spp. and the most common serogroups isolated in the area. Moreover, the results of the research will provide the opportunity to carry out epidemiological surveillance in the event of a suspected case.

## 2. Materials and Methods

A total of 100 samples, from the showers of 25 private homes in the Prefecture of Arta, were collected. Some extra data was also collected through interviews with the inhabitants, concerning the below subjects:

1. the age of the properties,
2. the heating system (central or independent),
3. the age of the water heater,
4. the prospective plumbing changes,
5. the length of stay in residence (continual or at times),
6. the frequency of water leaks or disruption of the water supply,
7. the age and gender of the habitants,
8. the medical history of pneumonia.

### 2.1. Sample Collection

Water samples (500 mL) were collected in dark glass tubes, which had been sterilized at 180°C for 2 hours. Before the sterilization, 0.5 mL sodium thiosulfate had been added to the tubes, in order to inactivate the remaining chloride from previous use of the tubes. The tubes were transferred to the laboratory in isothermal coolers at temperature between 6°C–18°C [11, 12].

Concerning the cold water, two samples were collected from each shower faucet. The first sample was collected after the release of the first drops of the cold water (direct sample). The second was collected after 2 minutes of water flow (indirect sample) [12].

Two samples of hot water were also collected. The first was collected after the release of the first drops (direct sample) and the second after 1 minute of water flow, in order to reach the maximum temperature (indirect sample) [12]. Temperature, free chloride, and pH were measured in each sample.

### 2.2. Sample Concentrations and Standard Culture Method

Water samples were concentrated by membrane filtration (0.45 *μ*m pore-sized filter) and with vacuum. Each membrane was transferred in a sterilized tube with 10 mL Ringer's solution and was vortex-mixed for 2 minutes. To reduce contamination by other microorganisms, 2 mL of this suspension was heat-treated (50°C for 30 min in a water bath); 0.1 mL from heat-treated and untreated suspension each was spread on plates with defined *Legionella* agar medium GVPC. The plates were incubated at 37°C in a humidified environment with at least 2.5% CO_2_ for 10 days. The plates were examined every two or three days at the dissecting microscope. Suspected colonies with a mottled surface or an iridescent cut-glass appearance were counted from each sampling. Three of these were selected each time and were subcultured on buffered charcoal yeast extract (BCYE) agar with cysteine and charcoal yeast extract agar cysteine-free. Only colonies grown on BCYE were subsequently identified by an agglutination test [2, 7].

## 3. Results 

According to the data that was collected through the interviews with the inhabitants, 56% of the houses were more than 20 years old, 20% were from 10 to 20 years old, and 24% had been constructed in the last 10 years. All the properties had independent water heating system. There was not any mention of recent water leakage or disruption of the water supply. 52% of the properties had undergone some minor changes in their plumbing systems. 16% of the houses are inhabited only at times and half of them are inhabited by people over 50 years old. The research was completed in an interval of 9 months, from August to April.

### 3.1. Cold Shower

Concerning the direct cold shower, 36% of the samples had temperatures below 20°C whereas 64% of them had temperatures over 20°C. Concerning the indirect samples 40% of them had temperatures below 20°C and 60% were over 20°C (Table 1).

The pH of the samples was measured between 7.5 and >7.9. More specifically 4% had pH 7.5, 4% had pH 7.7, 12% had pH 7.8, 4% had pH 7.85, 20% had pH 7.9, and 56% had pH over 7.9 (Table 2).

Only three of all samples (8%) had residual chloride over 0.2 mg/L, a rate that is necessary for potable water according to the Greek hygienic practice, whereas 60% of the samples had residual chloride less than 0.2 mg/L.

*Legionella* spp was detected in 3 of the 50 samples of cold water, 2 of which were direct samples and 1 an indirect sample. In 1 of the 2 direct samples *Legionella* spp. was detected after the thermal treatment as well.* Legionella pneumophila* serogroup 2–14 was detected in 2 of the 3 samples, whereas in the third sample *Legionella anisa* was identified (Table 5).

### 3.2. Hot Shower

From the hot shower, 12% of the direct samples had temperatures of 20–35°C, 32% had temperature between 35–45°C, 52% had temperature between 45–60°C, and 4% had temperature over 60^*ο*^C. Concerning the indirect samples, the temperatures below were recorded: 35–45°C in 16% of the samples, 45–60°C in 64% of the samples, and over 60°C in 20% of the samples (Table 3).

Regarding the pH of the indirect samples, it was measured between 7.4 and 7.9. More particularly 4% of the samples had pH 7.4, 16% had pH 7.6, 16% had pH 7.7, 16% had pH 7.8, 40% had pH 7.9, and 8% had pH 7.85 (Table 4).

*Legionella* spp was detected in 3 of the 50 samples of hot water, 1 of which was a direct sample and 2 were indirect samples. In both indirect samples *Legionella *spp. was detected after the thermal treatment as well. *Legionella pneumophila* serogroup 2–14 was indentified in all 3 samples (Table 5).

## 4. Discussion

In our study, *Legionella* spp. was isolated in 12% of domestic water samples. Similar results were produced in a research, where tap water samples were taken in the homes of 65 hematooncologic patients who were discharged from the hospital. *Legionella* spp. was cultured from six of the households (9.2%) [13]. Likewise, in another study in Catalonia, *Legionella* spp. was isolated in 8% of the water samples from the houses of 124 patients with Legionnaires' disease [8].

*Legionella pneumophila* serogroup 2–14 was identified in 5 of the 6 positive samples of our research. The isolation of this serogroup provokes interest because of the fact that serogroup 1 predominate in environmental samples. In the research conducted in Catalonia *Legionella pn*. serogroup 1 was isolated in 6 from the 9 *Legionella *positive homes of the patients with Legionnaires' disease. The same serogroup was isolated in only 1 of the 6 positive control homes. In the other 5 houses serogroup 2–14 and other *Legionellae* were identified. In our research none of the participants had a medical history of pneumonia. These data could support previous findings, which defend the theory that *Legionella* serogroup 1 is most commonly linked to human disease [14]. It must be also mentioned that *Legionellae* species and serogroups differ concerning their ability to survive in variable water environments. In particular, *L. pneumophila* serogroup 1 shows a special ability to survive under more stressful conditions, such as higher temperatures and chloride levels, which are not consistent with the survival of other serogroups. This leads us to the conclusion that by using common practices, such as increasing the temperature of the water or often changing the showerheads, we could decrease the risk of water contamination by *Legionella *serogroup 2–14.

In 1 of our 6 positive samples the isolated species was *Legionella anisa. *It is necessary to keep in mind that contamination with *Legionella anisa *could sometimes mask a further contamination with *Legionella pneumophila* serogroup 1 [15, 16]. There are some possible reasons that could explain the above observation. One possible reason is that, if *Legionella anisa *contamination levels are high, despite careful observation of each suspect colony, the rarer *L*. *pneumophila* colonies may be masked. Another reason is that *Legionella pneumophila *is more resistant to chemical and thermal shock. Heat shock may therefore have a less successful effect on *L*. *pneumophila* than *L. anisa, *abolishing bacterial interference within samples and making it easier to detect *L*. *pneumophila* microbiologically. Moreover, the heat shock applied to the water system may have disrupted the biofilm, leading to the circulation of previously sessile bacteria [15].

Concerning the hot water, the mean temperature of the samples negative for *Legionella* was higher compared to the mean temperature of the positive for *Legionella* samples (49.9°C versus 45.5°C). These findings agree with research in Finland, where the mean water temperature after heating was slightly higher in the *Legionella-*negative systems than in the *Legionella*-positive systems (53.5°C versus 51.5°C), as well as in a research in Ohio, where lower water heater temperatures were associated with *Legionella* colonization [9, 17]. On the other hand, in an investigation in Italy the presence of* Legionella* was not affected by water temperature [7].

The residual chloride plays a very important role in the clearness of the water. The residual chloride detection of our samples showed that only three of all samples had over 0.2 mg/L, whereas 60% of the samples had residual chloride less than 0.2 mg/L. Research from the international bibliography reports that there is no detection of *Legionella* spp. when residual chloride is over 0.4 mg/L [18]. However, contrary results have been revealed by other studies, according to which *L*. *pneumophila* is repeatedly isolated from chlorinated water systems, indicating that this treatment is not effective at preventing colonisation [19].

In the research of Bates et al. who used the same diagnostic method as in our research, none of the 100 collected samples tested positive for *Legionella* spp. The same samples were examined with two other methods as well: the PCR and the DFA. The PCR method produced 12 samples positive for *Legionella* spp., of which 6 were positive with the DFA method as well [20]. Similar were the results from the research of Edagawa et al. where only 4 samples in a total of 130 gave positive results for *Legionella* spp. using the culture method, whereas 26 samples showed positive when the PCR method was used [21].

It also must be taken into account that when using PCR methods for recovery of microorganism from environmental samples, there is always a risk that the isolated DNA may not come from live microbial cells [22].

The samples collection took place between August and April, thus including all the seasons of the year. Although all the positive samples were collected in September and early October, seasons that are usually linked to Legionnaires' disease, it is difficult to talk about seasonal variability of water contamination, since the research did not proceed to a second cross-checking collection of the samples during winter. The above pattern is thought to reflect meteorological factors such as increased temperature and humidity observed during the summer months and early autumn [23]. Additionally, according to research of domestic water tap in north-central United States (USA) for free-living amoebae, Vahlkampfia and Naegleria were the amoebae detected mainly in the autumn [24].

Recent studies have shown that contamination of the water is stable all year long, concerning not only the species of *Legionella *isolated, but also the concentration as well [17].

Our observations suggest that *Legionella* species should be considered when examining environmental contamination, which is essential to better evaluate environmental risk factors and select the most appropriate prevention and control measures. To limit *Legionella *colonization at the domestic level, we suggest simple and general measures: (1) maintaining high cleaning standards, (2) increasing the temperature of the water, and (3) periodically replacing components of the system which could favor presence or dissemination of bacteria.

## 5. Conclusions

According to our research, it is estimated that there is risk of infection through the use of showers, taking into consideration that two of the three houses, where the *Legionella* was detected, are inhabited by elderly people with other chronic conditions.

The results of our study agree with the research of Velonakis et al. in Greece, where *Legionella* spp. has been isolated from other Prefectures as well (unpublished data).

The temperature, which was found not only within the limits favorable to developing *Legionella* spp. (20–45°C) but also within the highest risk range (35–45°C) of the ideal zone for developing *Legionella* spp., and the low concentration of chloride provide fertile ground for development and proliferation of the bacteria, and other microorganisms as well.

## Figures and Tables

**Table 1:** Temperature of cold shower.

Temperature	Direct cold shower	Indirect cold shower
<20°C	36%	40%
>20°C	64%	60%

**Table 2:** pH of cold shower (indirect samples).

pH	Percentage
7.5	4%
7.7	4%
7.8	12%
7.85	4%
7.9	20%
>7.9	56%

**Table 3:** Temperature of hot shower.

Temperature	Direct hot shower	Indirect hot shower
20–35°C	12%	0%
35–45°C	32%	16%
45–60°C	52%	64%
>60°C	4%	

**Table 4:** pH of hot shower (indirect samples).

pH	Percentage
7.4	4%
7.6	16%
7.7	16%
7.8	16%
7.85	8%
7.9	40%

**Table 5:** Results of the culture for *Legionella* spp.

	Type of sample	Number of colonies	Type of *legionella *spp.
Resident 1	Direct hot sample	4000 cfu/L	*L. pn *2–14
	Direct hot sample (after thermal treatment)	4000 cfu/L	*L. pn* 2–14
	Indirect hot sample	3500 cfu/L	*L. pn *2–14
	Indirect hot samples (after thermal treatment)	2500 cfu/L	*L. pn *2–14

Resident 2	Direct cold sample	500 cfu/L	*L. pn *2–14
	Direct cold sample (after thermal treatment)	1000 cfu/L	*L. pn *2–14
	Indirect cold sample	1500 cfu/L	*L. pn *2–14
	Indirect hot sample	500 cfu/L	*L. pn *2–14
	Indirect hot sample (after thermal treatment)	1000 cfu/L	*L. pn *2–14

Resident 3	Direct cold sample	500 cfu/L	*L. anisa *
